# Shifting the HIV Paradigm from Care to Cure: Proceedings from the Caribbean Expert Summit in Barbados, August 2017

**DOI:** 10.1089/aid.2017.0310

**Published:** 2018-07-01

**Authors:** R. Clive Landis, E. Akinola Abayomi, Brendan C. Bain, Edward Greene, George Janossy, Patrice Joseph, Deanna Kerrigan, J. Philip McCoy, Cesar Nunez, Maurice O'Gorman, Alexander Pastoors, Bharat S. Parekh, Kim R. Quimby, Thomas C. Quinn, Kevin R. Robertson, Réjean Thomas, Eric van Gorp, Sten H. Vermund, Valerie Wilson

**Affiliations:** ^1^ Edmund Cohen Laboratory for Vascular Research, George Alleyne Chronic Disease Research Centre, The University of the West Indies, Bridgetown, Barbados.; ^2^ Office of the Deputy Principal, The University of the West Indies - Cave Hill Campus, Bridgetown, Barbados.; ^3^ Division of Haematopathology, Faculty of Medicine, Tygerberg Academic Hospital, Stellenbosch University, Cape Town, South Africa.; ^4^ Department of Medicine, University Hospital of the West Indies, Mona, Jamaica.; ^5^ Office of the UN Secretary General, United Nations, New York, New York.; ^6^ Department of Immunology, University College Medical School, University College, London, United Kingdom.; ^7^ Groupe Haïtien Etude pour le Sarcome de Kaposi et les Infections Opportunistes (GHESKIO), Port-au-Prince, Haiti.; ^8^ Department of Health, Behavior and Society, The Johns Hopkins Bloomberg School of Public Health, Johns Hopkins University, Baltimore, Maryland.; ^9^ National Heart, Lung, and Blood Institute, National Institutes of Health, Bethesda, Maryland.; ^10^ UNAIDS Latin American and Caribbean Regional Support Team, Panama City, Panama.; ^11^ Department of Pathology and Laboratory Medicine, Children's Hospital Los Angeles, Keck School of Medicine, University of Southern California, Los Angeles, California.; ^12^ Dutch Association of PLHIV, Amsterdam, the Netherlands.; ^13^ Division of Global HIV and TB, Centers for Disease Control and Prevention, Atlanta, Georgia.; ^14^ National Institute of Allergy and Infectious Diseases, National Institutes of Health, Bethesda, Maryland.; ^15^ Department of Neurology, University of North Carolina, Chapel Hill, North Carolina.; ^16^ Clinique médicale l'Actuel, Montreal, Canada.; ^17^ Department of Viroscience, Erasmus Medical Centre, Rotterdam, the Netherlands.; ^18^ Yale School of Public Health, New Haven, Connecticut.; ^19^ Caribbean Med Labs Foundation, Port of Spain, Trinidad.

**Keywords:** HIV, treatment, prevention, cure, stigma, monitoring

## Abstract

The CCAS EXPERT SUMMIT convened an array of international experts in Barbados on August 27–31, 2017 under the theme “From Care to Cure–Shifting the HIV Paradigm.” The Caribbean Cytometry & Analytical Society (CCAS) partnered with the Joint United Nations Programme on HIV/AIDS (UNAIDS) to deliver a program that reviewed the advances in antiretroviral therapy and the public health benefits accruing from treatment as prevention. Particular emphasis was placed on reexamining stigma and discrimination through a critical appraisal of whether public health messaging and advocacy had kept pace with the advances in medicine. Persistent fear of HIV driving discriminatory behavior was widely reported in different regions and sectors, including the healthcare profession itself; continued fear of the disease was starkly misaligned with the successes of new medical treatments and progress toward the UNAIDS 90-90-90 targets. The summit therefore adopted the mantra “Test-Treat-Defeat” to help engage with the public in a spirit of optimism aimed at creating a more conducive environment for persons to be tested and treated and, thereby, help reduce HIV disease and stigma at the individual and community levels.

## Introduction

The Caribbean Cytometry & Analytical Society (CCAS) EXPERT SUMMIT “From Care to Cure—Shifting the HIV Paradigm” reviewed advances in HIV diagnostics, treatment, prevention, and public health policy that have collectively shifted the paradigm from care to cure. The Expert Summit sessions (August 27–29, 2018) were followed by sessions on Caribbean issues (August 30 and 31, 2018). The whole program was delivered in plenary forum to encourage rich discussion among presenters and delegates, from as many backgrounds and viewpoints as possible (see Supplementary Data for full program; Supplementary Data are available online at www.liebertpub.com/aid).

Attendees were drawn from 25 countries with diverse backgrounds, representing roughly equal proportions of: medical professionals; natural scientists; and social scientists/community representatives. The program also examined challenges in areas of stigma, discrimination, gender, linkage to care, and biosecurity that continue to frustrate medical and policy gains.

## HIV Diagnostics

The summit heard from **Phil McCoy** (National Institutes of Health) on the historical impact the AIDS epidemic had on the clinical flow cytometry industry. He explained that clinical demand for CD4^+^ cell testing acted as the tipping point for the industry in the 1980s, transforming it from a mainly technology driven to an application driven sector.^1,2^ Over the ensuing decades, miniaturization and simplification of equipment and improvements in reagent stability were mostly driven by the needs and limited resources of developing countries where the AIDS epidemic was centered.^3^

**George Janossy** (University College London) picked up on this theme, reiterating how HIV diagnostics had created a new paradigm in international development envisaging bidirectional “codevelopment” and “reverse innovation” between North and South.^4^ He cited the “Afford CD4” North–South collaborative as a best practice for technological cocreation, which drove the expansion of clinical flow cytometry and user-friendly external quality assurance programs such as Quality Assessment and Standardization for Immunological Measures and National External Quality Assessment Schemes,^5^ also reaching point-of-care settings.^6^ This required simplification of guidelines for CD4 enumeration from dual to single platform, with developing countries adopting the guideline revisions. An example of reverse innovation was noted with the Pan-LeucoGating (PLG) protocol, developed at the University of Witwatersrand and subsequently rolled out into routine CD4 diagnostics worldwide.^7^

The U.S. Centers for Disease Control and Prevention (CDC) has played a key role in scaling up laboratory point-of-care testing under the President's Emergency Program For AIDS Relief (PEPFAR). **Bharat Parekh** (CDC) reported that some 74 million HIV rapid tests were conducted in 2016 at PEPFAR supported sites, and this has required a corresponding scale up of technical support to assure quality at remote testing sites.^8^ CDC has developed multiple quality assurance tools for rapid testing with the main pillar of support provided by the dried tube specimen (DTS) proficiency testing panels that are designed for external quality assurance in a real-world setting. DTS-based panels now include HIV serology, HIV viral load, syphilis, malaria, and tuberculosis.^9^

A repeated mantra at the Summit was “Treat Early, Treat All.” This requires early detection of HIV and ideally a knowledge of the time elapsed since seroconversion. Parekh discussed a newly developed “Asanté™ HIV-1 Rapid Recency” assay,^10,11^ currently approved for research purposes. The assay uses a chimeric p41 protein in trace amounts to detect only high avidity antibody and is capable of categorizing recency of HIV infection into binary events: seroconversion <6 months or >6 months. Accordingly, the rapid recency assay can diagnose HIV infection and detect recency of infection in one test.

Phil McCoy indulged the summit with the very latest frontiers of diagnostic multiplex technology, as he introduced an instrument capable of reading samples at single-molecule resolution. The “Simoa HD-1™” analyzer (single molecule array) utilizes blue ray discs to house vast arrays of femtoliter wells containing immunobeads. The tiny reaction volumes eliminate diffusion limitations and boost sensitivity to the single molecule level, 1,000 × more sensitive than existing detection methods for HIV p24 antigen.^12^ Whether this ultrasensitive technique may be used for earlier detection of HIV seroconversion remains to be investigated.

## Treatment Advances

**Clinique médicale l'Actuel** has been at the forefront of HIV research and treatment since 1984 when it was founded by **Réjean Thomas** as the first dedicated sexually transmitted infection (STI) clinic in Canada.^13^ Réjean Thomas reported on trends in treatment at Clinique l'Actuel, which manages one of the oldest cohorts of patients living with HIV (>60% of patients aged over 50).^14^ He reminded the Summit that the “Treat All” paradigm had an echo in the “Hit Hard, Hit Early” campaign of the late 1990s, which was undermined by the toxicity and visibly stigmatizing side effects (i.e., lipodystrophy) of the medication available at that time.^15^

The guiding principle of treatment today at Clinique l'Actuel is to keep it “light and simple.” The clinic is therefore shifting away from using protease inhibitors and is starting therapy with integrase inhibitors. With an aging patient population, Clinique l'Actuel is also turning to tenofovir alafenamide (TAF) instead of tenofovir disoproxil fumarate (TDF) to avoid side effects on bone and kidney, while efavirenz is used at lower dosage (400 mg) to minimize neurological complications.^16^ The clinic functions as a one-stop shop to monitor other STIs, such as syphilis, and also incorporates a laboratory and chronic disease clinic as part of a holistic approach to healthcare. The practice of using a one-stop clinic was also mentioned by **Patrice Joseph** (GHESKIO, Haiti) as a means of improving efficiency and ensuring contact with patients for purposes of monitoring care and treatment.^17^

The pre-exposure prophylaxis (PrEP) program at Clinique l'Actuel has enrolled over 1,400 patients since 2011. PrEP was initially offered to serodiscordant partners. It has since been extended to high risk men who have sex with men (MSM). Clients who are offered PrEP have a history of multiple partners (mean of 25.3 in the preceding 12 months), a history of antecedent STIs, and drug use (mostly cocaine and crystal meth).^18^ PrEP is offered either as a daily regimen or on demand based on client needs.

At Clinique l'Actuel, younger clients tended to prefer daily regimens to match sexual behavior that was more spontaneous and including multiple partners, whereas older clients were more likely to choose on-demand PrEP to match sexual behaviors that were more planned.^19^ All clients enrolled in the PrEP program received health education at mandatory consultations during enrollment and at clinic visits. Although the majority of current clinical guidelines recommend daily PrEP, the on-demand cohort at Clinique L'Actuel has reported zero seroconversions thus far among their 225 clients.

A best practice for HIV care and prevention in resource limited settings was presented by Patrice Joseph at the GHESKIO Center in Haiti. The GHESKIO Center is a not-for-profit organization that has been at the forefront of HIV care, treatment, and research from the outset of the epidemic in 1983.^20^ Their global health model of HIV care is notable for being evidence-led, with continuous cycles of research, publication, and improvement yielding remarkable health outcomes despite the adverse external environment and shortage of doctors in Haiti.^21^

Despite these constraints, GHESKIO manages the largest HIV and the largest TB clinics in the Americas, with 10-year survival outcomes for patients living with HIV on antiretroviral therapy (ART) similar to those in the United States.^22^ The shortage of doctors has prompted a search for more efficient and streamlined models of healthcare; nurse practitioners play a key role in this model, fast-tracking uncomplicated HIV patients through the clinic within 30 min, including dispensing of prepacked medication, leaving physicians free to deal with more complex cases.^23^ A same-day rapid pathway for HIV testing and initiation of ART has yielded further clinical improvements in terms of linkage to care, viral load suppression, and survival.^24^ The GHESKIO model is therefore a prime example of “reverse innovation”,^4^ wherein a best practice from a resource limited setting—impelled by those very limitations—can inform best practice everywhere.

**Kevin Robertson** (University of North Carolina) reviewed the impact of antiretroviral treatment from the perspective of the brain. Although the prevalence and severity of HIV Associated Neurological Disorder (HAND) have diminished since the introduction of ART, there remains concern for approximately one quarter of patients who still manifest mild-to-moderate HAND.^25^ The need to commence treatment immediately upon diagnosis was reinforced by the findings of a unique test and treat study carried out on incident HIV cases in a high risk MSM and transgender cohort in Peru.^26^ The ¿SABES? study, which had enrolled participants in the era before the START study findings were known, randomized acute and recent HIV cases to immediate ART versus 24-week deferment of ART.

¿SABES? demonstrated that in the immediate ART arm HAND was significantly improved at 48 weeks compared with the deferred arm, thereby recapitulating in the brain what the START and Temprano studies have demonstrated in the systemic compartment. The next phase of research on HAND is to investigate whether intensification of ART can tackle the residual impairment still noted in a minority of patients; this study is in progress: Integrase and Maraviroc Intensification in Neurocognitive Dysfunction (InMIND).^27^ Robertson pointed out that there may also be scope for combining ART with anti-inflammatory regimens, since inflammation is involved in the neuropathogenesis of HAND.

## Prevention

“Treatment as prevention” rightly begins as a concept with the prevention of vertical HIV transmission from mother to child. From the mid 1990s, cohort studies were able to demonstrate significant reductions in maternal-to-infant transmission of HIV when mothers received antiretroviral monotherapy during pregnancy and/or delivery. These findings were rapidly adopted and scaled into global prevention of mother to child transmission (PMTCT) programs.

In contrast, the prevention of horizontal HIV transmission by sexual intercourse, based on the same principle, has been more difficult to embed into global prevention. Several discussions at the CCAS Expert Summit commented on the relative failure of the healthcare profession to articulate clearly what “treatment as prevention” actually means for the public, with a consequence that obtuse messaging may have played a part in perpetuating fear and stigmatization of a disease that is not warranted given the availability of effective treatment.

**Thomas Quinn** (NIH) presented a status update on “treatment as prevention” in 2017. He led the Rakai Study group in 2000 that was the first to demonstrate in an observational cohort that heterosexual HIV transmission did not occur between serodiscordant couples at viral loads <1,000.^28^ A contemporaneous Zambian partner study was similarly designed and found similar results.^29^ It took another decade until a randomized clinical trial—the HIV Prevention Trials Network (HPTN) 052 study—was able to confirm that heterosexual transmission of HIV was eliminated in persons with suppressed viral load,^30^ and a further 5 years to establish the same for men who have sex with men in the PARTNER study.^31^

**Sten Vermund** (Yale University) highlighted the confluence of personal and public health benefits of treatment as prevention in the 10-year follow-up to HPTN 052. That study reported that the prevention effect had been maintained at 93% for index-linked transmissions and that no transmission had occurred when viral suppression was maintained.^32^ Personal health benefits in the early treatment arm included fewer AIDS clinical events and longer time to event,^33^ consistent with the findings of the START and Temprano trials.

Sten Vermund moved the discussion from the controlled environment of a randomized control study to the reality of carrying out treatment as prevention in the community. He described the PopulationART (PopART) HPTN 071 study, which is a cluster randomized trial conducted in 21 communities in Zambia and South Africa.^34–36^ Although it has not yet reported on primary prevention outcomes, the trial has already revealed some worrying gaps toward hitting the necessary HIV testing/treatment/suppression targets.^37^ One of the biggest problems encountered is a high level of mobility and migration recorded between successive rounds of community testing, such that new individuals who may not know their HIV status are continually joining the community and those with HIV may not receive primary care services.

Gender disparities were also noted, with males being under-represented during the first round of testing. However, innovative male clinics featuring interesting activities, not solely related to health, and scheduled at more convenient times on evenings and Saturdays, were able to achieve a marked turnaround in male enrollment.^37^ Such challenges at community level represent in microcosm the challenges faced in reaching the global 90-90-90 targets. The three pillars of the Joint United Nations Programme on HIV/AIDS (UNAIDS) 90-90-90 targets are: 90% of persons living with HIV who know their HIV status; 90% of persons living with HIV who know their status and are on ART medication; and 90% of persons on ART who have suppressed viral load.

Vermund emphasized that implementation science lies at the heart of rolling out treatment as prevention in the community and, hence, the ability to hit the three targets set by UNAIDS. Noting problems encountered in HPTN071 with high levels of migration and difficulties in recontacting persons between successive rounds of testing, he proposed that same-day HIV testing and initiation of ART must be part of the solution to addressing these gaps in linkage to care.

The Rapid Antiretroviral Program Initiative for new Diagnosis study in San Francisco was discussed by Thomas Quinn; this study demonstrated significantly shortened time to viral suppression in HIV patients who were prescribed ART medication on the same day as they tested positive for HIV, enabling the clinic to observe the first dosage and supply the patient with a pill-pack and follow-up appointment card.^38^ Quinn also commended an anonymous screening technique through Accident and Emergency departments to assist with epidemiological monitoring of community 90-90-90 targets.^39^ In this elegant approach, routine bloods collected for other purposes were analyzed through mass spectrometry and molecular diagnostics for the three pillars of prevention: HIV serostatus, presence of ART drugs, and viral suppression.

## Cure

**Cesar Nunez** (UNAIDS) explained that the purpose of the global 90-90-90 target was to reduce HIV transmission and illness to the point that HIV ceased to be a public health epidemic of international concern. While it may be too soon to claim a “cure” for HIV, Thomas Quinn pointed out that an estimated 9 million deaths had been averted by achieving the 2015 UN target to place 15 million persons living with HIV on ARTs.^40^ Further scale-up to meet the UNAIDS Fast Track target of 30 million persons on ARTs by 2020 also remained on target. Cesar Nunez captured the mood of optimism at the Summit, noting that the HIV epidemic had reached a tipping point in 2016: marking the first instance that a majority of persons living with HIV in the world (53%) were receiving ART medication.^41^

Some, but not all, mathematical models have predicted that when 90% of persons living with HIV know their HIV status, and 90% of persons living with HIV who know their status are on ART medication, and, in turn, 90% of persons on ART have suppressed viral load, then the HIV epidemic will no longer be able to sustain itself.^34,42^ The final 90-90-90 target therefore is 73% of persons living with HIV with suppressed viral load (0.90 × 0.90 × 0.90 = 0.73), although UNAIDS urges a 95-95-95 target by 2030.^43,44^ The Latin America and Caribbean (LAC) region was praised, alongside eastern and southern Africa, for being the first among the developing country blocs to achieve >40% viral suppression, thereby placing these regions on close par with the developed country bloc consisting of western and central Europe and North America.^41^

Among the LAC bloc, Cuba stood out for having almost reached the first 90% pillar and Cesar Nunez pointed out that the apparent spike in HIV cases recorded in Cuba was likely due to improvements in diagnosis. Haiti had achieved the milestone of reaching the second 90% pillar (placing persons on ART), while Brazil and Chile had achieved the third 90% pillar (viral suppression). So far, no country had achieved all three pillars. The predominant view from the Expert Summit was that the UNAIDS 90-90-90 and 95-95-95 targets had set ambitious goals that have galvanized countries to pursue the radical diminution of AIDS by 2030.

**Maurice O'Gorman** (UCLA) presented on the global PMTCT elimination targets and held the view that a generation of children without HIV was a realistic vision. Cuba had become the first country in 2015 certified by WHO to have eliminated mother to child transmission^45^; it has since been joined by Armenia, Belarus, Moldova, and Thailand.^46,47^ The UNAIDS elimination target is less than 50 babies born with HIV per 100,000 live births to mothers with HIV. The process target is to place >95% of pregnant mothers living with HIV on ARTs throughout pregnancy, delivery, and breastfeeding. Based on process targets, Maurice O'Gorman encouraged the Caribbean to set the ambition to become the first region in the world to eliminate vertical transmission of HIV from mother to child. His words were prophetic, as the following six Caribbean countries have been certified since the Expert Summit as having eliminated mother to child transmission of HIV and syphilis: Anguilla, Antigua and Barbuda, Bermuda, Cayman Islands, Montserrat, and St. Kitts and Nevis.^48^

Sten Vermund repeated the point that implementation science was key toward driving PMTCT outcomes at the community level. He cited a cluster randomized trial in rural north-central Nigeria that integrated PMTCT and maternal and child health services, allied with point-of-care diagnostics, to deliver greatly improved outcomes in HIV-positive mothers.^49^ The intervention recorded statistically significant improvements in: initiation of ART in HIV^+^ pregnant mothers, retention of mothers and children in care, and reduced prevalence of HIV-positive infants at 12 weeks of age (2.4% vs. 7.3%).

## Stigma

Despite the advances in medicine and the steady increase in ART coverage, Cesar Nunez warned that “the potential impact of game-changing scientific advances is being undermined by ignorance, fear, shame, prejudice, and exclusion.” He reported on an important survey conducted by UNAIDS among eight Caribbean countries between 2013 and 2016, which highlighted stubborn and widespread attitudes of fear and discrimination.^40^ For example, the leading barrier against a person wanting to be tested was identified as fear of being stigmatized if news of the result came out and concerns about confidentiality.^50^

Fear and stigma associated with HIV were identified by delegates at the Expert Summit as the primary driver of discriminatory behaviors reported from across the regions of the world.^40^ Even in a nominally liberal country such as The Netherlands, **Alexander Pastoors** (Dutch Association of people living with HIV) reported that deeply entrenched attitudes of fear and misinformation were fueling stigmatization of persons living with HIV.^51^

Perhaps most worrisome was that discriminatory behaviors were prevalent within the healthcare profession itself. Negative attitudes and unprofessional conduct towards persons living with HIV were recorded among a significant proportion of healthcare workers surveyed in the Caribbean.^40,50^ Similar barriers were noted within the healthcare system of the Dominican Republic by **Deanna Kerrigan** (Johns Hopkins University) during her CCAS 2017 Distinguished Lecture on the “Abriendo Puertas” (Open Doors) program.^52^

Abriendo Puertas is a community-driven multilevel intervention among HIV^+^ female sex workers (FSWs) in the Dominican Republic. The intervention revealed that parts of the healthcare system were difficult to access among FSW patients in the Abriendo Puertas program and that peer navigators were needed to help patients gain access to health services and counteract discriminating behaviors by the healthcare profession. The same issue of heightened stigmatization within the healthcare setting was identified in the Netherlands by Alexander Pastoors. He asserted that “living with stigma” was now a worse problem than “living with HIV” in the era of ART.

Pastoors commended the “Seven Es” program of the Dutch Association of PLHIV to tackle stigma and misinformation (Table 1). He remained especially concerned that vague health messaging surrounding treatment as prevention was inadvertently perpetuating stigmatization (and self-stigmatization) of persons living with HIV. The #UequalsU campaign (Undetectable = Uninfectious) makes the valid point that: *“All people living with HIV have a right to accurate and meaningful information about their social, sexual, and reproductive health”.*^53^ Should the truth about treatment as prevention—that persons with undetectable viral load are uninfectious—be viewed as a fundamental human right?

**Table 1.** “Seven Es” Program of the Dutch Association of PLHIV

Educate	Healthcare professionals
Enable	PrEP
Emancipate	Patients (to make informed decisions about their health, decide when and to whom to disclose, and cope with stigma)
Engage	With other communities and peers
Encourage	Disclosure (to select persons at least)
Enlighten	The public (to debunk myths)
Endorse	Destigmatizing messages (exemplified by #UequalsU)

PLHIV, people living with HIV; PrEP, pre-exposure prophylaxis.

**Clive Landis** (University of the West Indies, Barbados) expanded on the notion that clearer health messaging on treatment as prevention should be viewed as an opportunity to martial support among the public to help eliminate HIV, through the theory of rational self-interest. He pointed out that the stigma associated with the disease, the fear of being tested, and even discriminating attitudes by members of the public could be explained as rational *IF* they were based on the false premise that HIV was a death sentence. On that premise, why *would* you seek to get tested? When the outcome is merely to be issued a death sentence. It therefore becomes essential to correct the widespread and persistent misapprehension—two decades after the advent of highly active antiretroviral therapy—that HIV is untreatable and equivalent to a death sentence.^54–56^

According to the theory of rational egoism, Landis argued, when you understand that a person on ART with suppressed viral load is noninfectious, then it becomes in your own rational self-interest to encourage HIV-infected persons to seek treatment. Conversely, it becomes irrational and harmful to your own self-interest to create barriers through stigmatizing attitudes against persons seeking testing or treatment. Landis led two plenary discussions with participation from delegates representing a wide range of perspectives and backgrounds to find a more engaging public health message to dispel some of the pessimism in the public discourse.

The Expert Summit endorsed the mantra: “Test-Treat-Defeat to align with the three pillars of 90-90-90.” The mantra is intended to appeal at a social and an individual level, to help create a more supportive environment in which persons can feel comfortable to come forward for testing and treatment and, hence, to defeat HIV.^57,58^ The mantra has been adopted at a regional level since the Expert Summit by the Pan Caribbean Partnership against HIV/AIDS (PANCAP), the mechanism responsible for coordinating the Caribbean regional response to HIV. “Test-Treat-Defeat” is included in Article II of a “Declaration of Commitment by PANCAP Champions for Change to contribute to fast track the end of AIDS in the Caribbean by 2030,”^59^ and it is published in the revised PANCAP Regional Advocacy Strategy.^60^

**Edward Greene** (United Nations Secretary-General's Special Envoy for AIDS in the Caribbean) noted that the “Test-Treat-Defeat” mantra was entirely consonant with UN Sustainable Development Goal 3.8 and the WHO Director General's statement that “All Roads Lead to Universal Health Coverage.” In his address to the Champions For Change meeting—subsequent to the Expert Summit—the UN Special Envoy used “Test-Treat-Defeat” as an umbrella message to promote social protection, social justice, access to public health as a human right, and fostering an enabling environment.^61^ However, Greene cautioned the Expert Summit that Universal Health Coverage will remain an elusive goal if stigma against persons living with HIV persisted within the healthcare profession.

He therefore welcomed analysis to better understand and address fears of the disease that may be underpinning discriminatory attitudes and agreed that the time had come to craft a more optimistic message on HIV to galvanize community support. Greene cited examples in the Caribbean where common ground had been found through the PANCAP Justice for All Programme between communities, such as the LGBT community and Caribbean faith leaders, based on a shared sense of purpose and optimism to eliminate AIDS.^62^ He also praised the leadership of the Caribbean first ladies in promoting the “Every Caribbean Woman, Every Caribbean Child” initiative, which he noted was ably supported by the men and boys in their respective “He4She” campaign. Greene commended such models of collaboration between the sexes and communities as exemplars of the UNAIDS/Global Solidarity Model to accelerate action for access.

## Gender

In her CCAS distinguished lecture, Deanna Kerrigan revealed the vulnerabilities and multiple stigmas faced by FSWs in the Dominican Republic. The majority of FSWs in the Abriendo Puertas program (93%) were mothers, with an average age of 36 years. Many were impelled to continue sex work to support their children.^63^ National statistics show that HIV prevalence in the Dominican Republic is six times greater in FSWs compared to women in the general population. A surprise finding was that the majority entered sex work *after* their HIV diagnosis. This paradoxical observation might be explained due to the illegal practice by employers of enforcing HIV testing among their staff, leading to dismissal of persons testing positive.^64^

Kerrigan reported that participants in the Abriendo Puertas program often experienced multiple forms of stigma related to being: (1) women, (2) poor, (3) sex workers, and (4) HIV^+^, including both in the community and in healthcare settings. The experience of Abriendo Puertas illustrates the need to reorientate healthcare systems from a provider-centered ethos toward being patient- and client centered. Clive Landis noted that such a reorientation had been achieved in the national HIV Clinic of Barbados and that this was accompanied by corresponding improvements in viral suppression and quality of life among clients attending the clinic, equivalent to levels in developed countries.^65^

## Monitoring

The theme of laboratory capacity building for the strengthening of monitoring and other critical programs in the Caribbean was presented by **Valerie Wilson** (Caribbean Med Labs Foundation, Trinidad). Wilson noted that the HIV epidemic had played a beneficial role in driving the regional laboratory strengthening agenda, even beyond HIV.^66–68^ She commended the concerted and coordinated approach shown by the international and regional organizations in the Caribbean to promote national laboratories and referral networks, involving collaboration between the Pan American Health Organization/World Health Organization (PAHO/WHO), the CDC, the Caribbean Public Health Authority (CARPHA), the Caribbean MedLabs Foundation (CMLF), and the CCAS.

Valerie Wilson presented a 9-year retrospective analysis of the Caribbean laboratory landscape, using data contained in Caribbean country reports submitted to the annual CCAS conferences from 2008 to 2016. The analysis revealed steady growth in HIV laboratory and monitoring infrastructure across the region, especially for indicators of molecular diagnostics, treatment cascades, governance (national policies and licensure), and accreditation (Table 2). Despite these gains in laboratory and monitoring infrastructure, concern was voiced by Edward Greene, Cesar Nunez, Sten Vermund, and other delegates that dwindling international funding might place at risk programmatic gains made in the Caribbean, as well as other developing countries, toward the 2030 UNAIDS Elimination Targets.

**Table 2.** Indicators of Caribbean Laboratory and Monitoring Infrastructure

*Indicator*	*2008 (*n = *12)*^a^	*2009 (*n = *14)*	*2010 (*n = *11)*	*2011 (*n = *10)*	*2012 (*n = *15)*	*2013 (*n = *11)*	*2014 (*n = *8)*	*2015 (*n = *15)*	*2016 (*n = *12)*
Demographic data	12^b^	14	11	10	13	11	7	15	12
ARV provided	12	14	11	10	13	10	7	15	12
PMTCT	12	13	9	10	13	10	7	15	12
PITC/VCT	11	14	10	10	12	9	7	15	12
CD4	12	13	11	10	13	11	7	15	11
VL	10	10	11	10	13	11	6	13	10
EID	1	10	9	9	8	7	4	6	11
HIV DR	0	2	5	4	6	5	3	3	5
Treatment cascade	0	0	0	0	0	0	2	14	12
Laboratory indicator report	0	0	0	0	0	8	2	13	11
National laboratory policy	0	0	0	0	0	0	0	11	8
Accredited HIV Ref laboratory	0	0	1	1	1	2	2	2	2

^a^ Total number of Caribbean countries reporting at the annual CCAS conference.

^b^ Number of Caribbean countries reporting compliance for any given indicator. Shading on a 10-point gray scale denotes the proportion of countries compliant for an indicator (Black = 100% of countries compliant; White = zero countries compliant).

ARV, antiretroviral therapy; CCAS, Caribbean Cytometry & Analytical Society; PMTCT, prevention of mother to child transmission; VCT/PITC, voluntary testing and counseling/provider initiated testing and counseling; CD4, flow cytometric enumeration of CD4 lymphocytes; VL, HIV-1 viral load by real-time PCR; EID, early infant diagnosis of HIV-1 infection by DNA-PCR; HIV DR, HIV drug resistance testing.

The Expert Summit was unanimous in the recommendation that whereas donor agencies reserved the right to change funding priorities as they saw fit, every effort should be made to leave intact funding support for the monitoring frameworks. This would allow the impact of funding decisions to be monitored objectively and is especially true for HIV viral load monitoring, as the essential metric for tracking 90-90-90 targets.^67^ Several of the smaller islands in the Caribbean, that are not equipped to conduct molecular diagnostics, have complained that HIV viral load monitoring through established laboratory referral networks is no longer being supported through international funding partners.^69^

**Akin Abayomi** (University of Stellenbosch, South Africa) addressed the related topics of bioethics and biosecurity. He argued that the original species jump of HIV-1 M serotype from simian SIV-1 in the era between 1915 and 1941 may have been triggered by a notorious bioethical accident linked to the illegal slave colonies and medical practices imposed on laborers in the Belgian Congo.^70–72^ The rise in emerging diseases in the present day may have a bioethical echo with indentured labor practices, by the exploitation and movement of impoverished work forces on a global scale.^73,74^ The health threat from HIV and emerging viruses has illustrated how bioethics and biosecurity are inextricably linked, and vigilance is needed to strengthen both.^75,76^

**Eric van Gorp** (Erasmus Medical Center, The Netherlands) supported Abayomi in urging governments to adopt biosecurity agendas as a matter of priority to prepare in a more systematic way for the inevitable next emerging disease outbreak.^77,78^ He pointed out that the Caribbean was especially vulnerable to emerging diseases because of its tropical location in the mosquito belt and travel destination as a tourism hub.^79^ He noted that recent Chikungunya and Zika epidemics, as well as the threat from Ebola, had exposed gaps in Caribbean preparedness in the areas of diagnostics, biosafety, quarantine-, and critical care facilities.^75^

Similar gaps and reactionary public health measures bordering on panic had manifested themselves across the world during the Ebola outbreak, even in developed countries.^76^ These realities have emphasized the need to enact a more systematic and measured response based on national biosecurity agendas in preparing for, and handling outbreaks of, emerging diseases.

## Conclusion

The CCAS Expert Summit was able to draw on expert opinion and research from different regions and across disciplines to report broadly positive movement toward a shifting HIV paradigm. The UNAIDS 90-90-90 targets had particularly galvanized countries to strive for elimination targets, with some countries already attaining elimination of mother to child transmission. Medication advances and earlier treatment—that logically extends to same-day testing and initiation of ART—are delivering improved clinical outcomes and, for the first time, a majority of persons living with HIV are receiving ART since the year 2016. Widening ART coverage and expansion of PrEP are also delivering prevention gains on sexual transmission of HIV.

This sense of progress and optimism, however, is not shared among the wider public—and even certain pockets of the healthcare profession—in which lingering fear and misinformation propagate stigma and discrimination toward persons living with HIV. To counter such misinformation, persons living with HIV are demanding as a human right more accurate information about the effect of ART on their own bodies. The Expert Summit supported clearer public health messaging and adopted the mantra “Test, Treat, Defeat” to capture the mood of optimism and role played by treatment as prevention on the road toward elimination of HIV. The organizers look forward to the next CCAS EXPERT SUMMIT in 2018.

## Supplementary Material

Supplemental data
