# Supplementary material for: Shifting the HIV Paradigm from Care to Cure: Proceedings from the Caribbean Expert Summit in Barbados, August 2017
Source: AIDS Res Hum Retroviruses. 2018 Jul 1;34(7):561–9. doi: 10.1089/aid.2017.0310 (PMC6053839; doi:10.1089/aid.2017.0310)
Supplement: Supplemental data [file Supp_Data.pdf]

Supplementary Data

CCAS EXPERT SUMMIT: From Care to Cure—Shifting the HIV Paradigm
Almond Beach Hotel, St. Peter, Barbados
August 27–31, 2017
Sunday August 27, 2017
Opening Ceremony

Master of Ceremonies: Vera Layne, President, Caribbean Cytometry & Analytical Society (CCAS)
6:30 pm: National Anthem of Barbados
6:35 pm: Opening Prayer
6:40 pm: Welcome: Clive Landis, Chairman, CCAS, Barbados
6:50 pm: Greetings: Barbados Ministry of Health—Anton Best
7:00 pm: Remarks: UNAIDS Latin America and the Caribbean—Cesar Nunez
7:10 pm: CCAS DISTINGUISHED LECTURE: Social and economic vulnerability of women: a nexus of risk—Deanna Kerrigan, Baltimore, USA
7:50 pm: Vote of Thanks: Valerie Wilson, Caribbean MedLabs Foundation
8:00 pm: Entertainment/cocktails

Monday August 28, 2017
Expert Summit Session 1
Chair: Clive Landis
8:30 am: A bumpy journey: from lipodystrophy to PrEP—Réjean Thomas, Montreal, Canada
9:15 am: CD4: a marker in time—George Janossy, London, United Kingdom

Coffee break 10:00–10:30 am
Expert Summit Session 2
Chair: Eric van Gorp
10:30 am: An explosion of technology innovation … driven from the South—Phil McCoy, NIH, USA
11:15 am: Quality diagnostics at the point of care—Bharat Parekh, CDC, USA

Lunch 12:00–1:00 pm
Expert Summit Session 3
Chair: Réjean Thomas
1:00 pm: Treatment as prevention: is anyone watching?—Thomas Quinn, Baltimore, USA
1:45 pm: Treatment as prevention a decade later: "The scientific discovery of the year"—Sten Vermund, Nashville, USA

Tea break 2:30–3:00 am
Expert Summit Session 4
Chair: George Janossy
3:00 pm: A generation of children without HIV: dream or reality?—Mo O'Gorman, Los Angeles, USA
3:45 pm: An opportunity to recast the HIV message on stigma and discrimination: the self-interest theory—Clive Landis, The University of the West Indies, Barbados

Tuesday August 29, 2017
Expert Summit Session 5
Chair: Mo O'Gorman
8:30 am: Mind over matter: challenges in neurocognition—Kevin Robertson, Chapel Hill, USA
9:15 am: Against all odds: Haiti leads the way—Patrice Joseph, Port-au-Prince, Haiti

Coffee break 10:00–10:30 am
Expert Summit Session 6
Chair: Patrice Joseph
10:30 am: Achieving the 90-90-90 targets in Latin America and the Caribbean progress and challenges—Cesar Núñez, UNAIDS
11:15 am: Stigma, the greatest hurdle to ending AIDS—Alexander Pastoors, Dutch Association of PLHIV, The Netherlands

Lunch 12:00–1:00 pm
Expert Summit Session 7
Chair: Thomas Quinn
1:00 pm: Ten-year retrospective on HIV capacity building in the Caribbean—Valerie Wilson, Trinidad
1:45 pm: What is the point? A cure without access—Edward Greene, United Nations

Tea break 2:30–3:00 pm
Expert Summit Session 8
Chair: Sten Vermund
3:00 pm: Care versus cure: different pathways for zika and HIV—Eric van Gorp, Rotterdam, Holland
3:45 pm: From care to cure: has the paradigm shifted?—Akin Abayomi, Cape Town, South Africa

5:00 pm End of Day 2
Wednesday August 30, 2017
Caribbean Country Reports
Chair: Valerie Wilson

8.30–10.00 am
Coffee break 10:00–10:30 am
Caribbean country reports … continued
Chair: Valerie Wilson

10.30–12.00 am
Lunch 12:00–1:00 pm
Research in the Caribbean
Chair: Eddie Greene
1:00 pm: A Single Thread—Jamonn Roberts, Revolt Visuals, Barbados
1:30 pm NCDs in PLHIV, their Care and Treatment: a Public Health Concern—Carmeta Doughlin, The University of the West Indies, Barbados
2:00 pm: The Barbados Aging with HIV Study—Damani Piggott, Johns Hopkins University, Maryland

Tea Break 2:30–3:00 pm
3:00: Industry sponsored talks (not part of CME program):
Chair: Phil McCoy
Alere
Beckton Dickinson
Beckman Coulter
Roche—"The Challenges You Face Evolve. So Stay One Step Ahead"
Ryvex—"Accurate Results to treat opportunistic infections"
Sysmex

4:30 pm: End of Day 3

Thursday August 31, 2017

Plenary Session: Violence and vulnerability of women in the Caribbean

Chair: Kevin Robertson

8:30 am: ''Governing'' violence: language, ''colonial'' gender, and change—Hamilah DeShong, The University of the West Indies, Barbados

9:00 am: The personal experiences and opinions of the girls from the Government Industrial School of Barbados: a qualitative study—Jacoline Bergman, Rotterdam University, The Netherlands

9.30 am: Correlates of sex work stigma among female sex workers living with HIV in the Dominican Republic—Tahilin Karver, Johns Hopkins Bloomberg School of Public Health, Maryland

Coffee break 10:00–10:30 am

Plenary Session: Research in Latin America and the Caribbean

Chair: Brendan Bain

10:30 am: Guyana key population size estimation validation—Nastassia Rambarran, Society against sexual orientation discrimination, Guyana

11:00 am: Urbanization, the Built Environment, and the Sexual Geography of HIV/AIDS—Paul Burns, Washington

11:30 am: Adapting a multilevel intervention to promote HIV prevention, care, and treatment outcomes for Transgender women sex workers in the Dominican Republic—Martha Perez, Unidad de Vacunas e Investigacion at Instituto Dermatologico y Cirugia de Piel, Dominican Republic

Lunch 12:00–1:00 pm

Plenary Session: Research in Latin America and the Caribbean, continued

Chair: Akin Abayomi

1:00 pm: Integrating substance use support and referral into a multilevel intervention for female sex workers living with HIV in the Dominican Republic—Hoisex Gomez, Unidad de Vacunas e Investigacion at Instituto Dermatologico y Cirugia de Piel, Dominican Republic

1:30 pm: ''Son unas perras, eso es lo que dicen'': a qualitative study of sex work stigma among women living with HIV in the Dominican Republic—Clare Barrington, University of North Carolina

2:00 pm: Abriendo Puertas: Scaling up a multilevel intervention for female sex workers living with HIV in Santo Domingo, Dominican Republic—Yeycy Donastorg, Unidad de Vacunas e Investigacion at Instituto Dermatologico y Cirugia de Piel, Dominican Republic

Tea break 2:30–3:00 pm

3:00 pm: Review and the way forward—Brendan Bain, University Hospital of the West Indies, Jamaica

4:00 pm: End of Day 4
